
JOURNAL INFORMATION
==============================
NLM Title Abbreviation: Inter Econ
Journal ID: Inter Econ
NLM Journal ID: 101086399

Inter Economics

ISSN: 0020-5346

ARTICLE INFORMATION
==============================
PMCID: PMC7704582
PMID: 33281208
DOI: 10.1007/s10272-020-0926-9
Article ID: 926
Article version: 1
Subjects: Editorial

The EU’s Pandemic Response: Tackling COVID-19, Building the Future

Gentiloni Paolo simon.o'connor@ec.europa.eu

grid.270680.b European Commission, Rue de la Loi / Wetstraat 200, 1049 Brussels, Belgium
Electronic publication date: 2020 Dec 1
Print publication date: 2020
Volume: 55
Issue: 6
First page: 342
Last page: 343
Copyright: © The Author(s) 2020
License: This article is distributed under the terms of the Creative Commons Attribution 4.0 International License (https://creativecommons.org/licenses/by/4.0/).
Open Access funding provided by ZBW — Leibniz Information Centre for Economics.
License URL: https://creativecommons.org/licenses/by/4.0/

issue-copyright-statement: © The Author(s) 2020

==============================

Any misplaced complacency there may have been during the summer that the public health crisis was behind us has been shattered by the developments of the last few weeks. Europe is now experiencing a strong second wave of the coronavirus pandemic, with numbers rising dramatically across our continent. Many countries that escaped relatively lightly in the spring have recently seen an alarming rise in cases, hospitalisations and deaths. Health workers across Europe find themselves once again on the front lines of this struggle and deserve all our solidarity and admiration.

The pandemic is also causing human suffering through its economic implications. Despite a strong rebound of the European economy in the third quarter of 2020 after containment measures were eased, the resurgence of the pandemic has abruptly interrupted our recovery. The Commission's recent Autumn Economic Forecast projects a decline in EU GDP of -7.4% in 2020, before bouncing back by 4.1% next year. This is the deepest recession in EU history, surpassing by far the financial crisis of 2009, when the European economy shrank by 4.5%. Yet, the downturn would have been much worse without the support measures adopted at national and European levels – about €3.5 trillion between fiscal and liquidity support and guarantees. Importantly, the figure for the EU as a whole masks an immense divergence across member states, from a decline of "only" -3.9% in Denmark to -12.4% in Spain.

It was clear already in March that the symmetric shock of COVID-19 risked provoking a very asymmetric crisis. Many of the countries that were hardest hit in the first wave were also those with the least fiscal space, with the most exposed sectors, and with lingering legacies of the previous crises. Europe is therefore facing a common challenge, requiring a common response, but one that needs to target its support where it is most required.

Of the first package of three measures decided in the Eurogroup before Easter, the first to become operational is the Support to mitigate Unemployment Risks in an Emergency (SURE) instrument. Over the last few weeks, the Commission has carried out two tremendously successful issuances of SURE social bonds for a combined value of €31 billion, and SURE payments to a number of member states have already taken place. These sales have been oversubscribed about 13 times and represent a strong vote of confidence in the EU's broader recovery plan, Next Generation EU.

At the heart of this agreement, reached in the European Council in July, is the Recovery and Resilience Facility that will provide up to €672 billion in grants and loans. This funding will be available to member states based on the implementation of 'Recovery and Resilience Plans'. As I am keen to stress, these plans will not be drawn up by the Commission but by the individual member states who will be responsible for the reforms and investments in question. The Commission's role will be to closely assess each plan submitted in order to make sure that it corresponds to the goals of the facility, namely to respond to the country-specific recommendations; to foster growth, job creation and resilience; and to contribute to the green and digital transitions.

This last point is crucial. Some feared that the coronavirus pandemic would lead Europe to retreat from its ambitious climate and sustainability commitments. The opposite is true. Not only have we remained committed to the European Green Deal as our growth strategy, but we have also strengthened our resolve and seized the opportunity offered by the recovery plan to put sustainability at the heart of our economic policy.

To begin with, the Commission has proposed to increase to 55% the reduction in emissions to be achieved in the European Union by 2030. Moreover, we have specified that of the financing to be made available to each member state through the Recovery and Resilience Facility, 37% should contribute to the green transition, with a further 20% for the digital transition. Furthermore, we have committed that 30% of the issuance that will fund Next Generation EU will be in green bonds. This comes on top of the total €100 billion of the issuance planned for SURE that will be through social bonds.

Next Generation EU can be one of the largest stimulus packages anywhere in the world, unleashing transformative investment and reforms in a host of key areas, from renewables and grid infrastructure to energy-efficient buildings and sustainable transport; from 5G and artificial intelligence to public and private sector digitalisation. Nevertheless, programmes for continuous learning, training and re-skilling will be equally important. The transition will need to be just, or it will just not happen.

As we turn to the implementation phase of Next Generation EU over the coming months, a key challenge for member states will be to absorb the immense sums made available fully and effectively. It will be crucial for each country to put a governance structure in place that ensures a strong central steering and coordination between the national and local level. The Commission will work in close partnership with our member states on this front, because this will be a test case for the Union as a whole. If we make a success of it, we will have laid the groundwork and provided a blueprint for how to tackle downturns in the future.

As the shadow of the pandemic lengthens once more over Europe, governments are once again having to make some difficult decisions. Shuttering restaurants, cafés, museums and theatres, restricting social contacts and physical movements – these measures are unfortunately necessary to flatten the curve of COVID-19 infections, even if we know they are likely to significantly slow economic activity in the fourth quarter of 2020.

There are two key policy implications from the latest developments: First, the co-legislators must redouble their efforts to close the deal on the Recovery and Resilience Facility without further delay. The sooner that happens, the sooner the ratification process can get underway, and the sooner Next Generation EU can begin to be implemented.

Second, we need to support the economy for as long as is needed. I believe that we have learned our lessons from the past crisis. Our general escape clause will remain in place throughout 2021 and as long as necessary. The discussion about how and when to normalise fiscal policy is one that I believe cannot be held in isolation from the discussion on how to improve our economic governance framework.

Next year, once Next Generation EU is up and running, the Commission will present its proposals in this area. Several interesting ideas have already been put forward, e.g. by the European Fiscal Board, such as the creation of a permanent fiscal capacity; mechanisms to facilitate investment, especially for the climate transition; and how to adapt our policies to reflect the reality of the much higher debt levels in a large number of member states after the pandemic.

This year, the EU's institutions and member states have collectively demonstrated a strong determination to act decisively together in very challenging circumstances. Through the long and difficult winter that lies ahead of us, we must maintain that determination. If we can do that, I am confident that the European Union can emerge strengthened from these challenging times.

Paolo Gentiloni, European Commissioner for the Economy, Brussels, Belgium.

